# *Streptococcus dysgalactiae* subsp. *equisimilis* Bacteremia, Finland, 1995–2004

**DOI:** 10.3201/eid1605.080803

**Published:** 2010-05

**Authors:** Sari Rantala, Susanna Vähäkuopus, Jaana Vuopio-Varkila, Risto Vuento, Jaana Syrjänen

**Affiliations:** Tampere University Hospital, Tampere, Finland (S. Rantala, R. Vuento, J. Syrjänen); National Institute for Health and Welfare, Helsinki, Finland (S. Vähäkuopus, J. Vuopio-Varkila); Medical School, University of Tampere, Tampere (J. Syrjänen)

**Keywords:** Bacteremia, beta-hemolytic, Streptococcus dysgalactiae subsp*.* equisimilis, emm type, streptococci, bacteria, Finland, dispatch, *Suggested citation for this article:* Rantala S, Vähäkuopus S, Vuopio-Varkila J, Vuento R, Syrjänen J. *Streptococcus dysgalactiae* subsp. *equisimilis* bacteremia, Finland, 1995–2004. Emerg Infect Dis [serial on the Internet]. 2010 May [*date cited*]. http://www.cdc.gov/EID/content/16/5/843.htm

## Abstract

We conducted a retrospective population-based study of 140 episodes of *Streptococcus* *dysgalactiae* subsp. *equisimilis* bacteremia occurring in Finland during 1995–2004. Rare *emm* types were associated with more severe disease and increased mortality rates. Skin and soft tissue infections were more frequent clinical signs among cases caused by common *emm* types.

Lancefield groups C and G β-hemolytic streptococci (GCS and GGS) may colonize the pharynx, skin, gastrointestinal tract, and female genitourinary tracts (*1*). According to recent taxonomic studies, large colony-forming groups C and G streptococci that infect humans are classified as *Streptococcus dysgalactiae* subsp. *equisimilis* (*2*). *S*. *dysgalactiae* subsp. *equisimilis* and *S*. *pyogenes* share virulence factors (*3*,*4*). The M protein is an important virulence factor because it confers resistance to phagocytosis (*5*). As with *emm* genes of *S*. *pyogenes*, the *emm* homologs of groups C and G *S*. *dysgalactiae* subsp. *equisimilis* are used for sequence-based typing (*4*,*6*,*7*), with >50 sequence types currently described (www.cdc.gov/ncidod/ biotech/strep/emmtypes.htm). The aim of our study was to determine the clinical signs, epidemiologic characteristics, and *emm* types of *S*. *dysgalactiae* subsp. *equisimilis* bacteremia during the 10-year observation period in Finland.

## The Study

We retrospectively reviewed the medical records of all adult patients (>16 years of age) in Pirkanmaa Health District, Finland, with >1 blood cultures positive for group C or group G *S.* *dysgalactiae* subsp. *equisimilis* from January 1995 through December 2004. The Pirkanmaa Health District (460,000 inhabitants) has 1 tertiary care hospital (Tampere University Hospital) and 4 other hospitals (Hatanpää City Hospital and the District Hospitals in Valkeakoski, Vammala, and Mänttä). Laboratory records were screened to identify all blood cultures positive for group C or group G *S.* *dysgalactiae* subsp. *equisimilis* during the study period. Our case definition included all patients who had a positive blood culture for *S*. *dysgalactiae* subsp. *equisimilis* and clinical signs compatible with septicemia. A severe disease was defined as a septicemia leading to death or needing intensive care unit treatment. All 128 GGS isolates and 12 of 18 GCS isolates were confirmed to be *S*. *dysgalactiae* subsp. *equisimilis*. Thus, these 140 episodes of *S. dysgalactiae* subsp. *equisimilis* septicemia (involving 137 patients) comprised the present study. Two of the isolates (1 GGS and 1 GCS) were not available for *emm* typing, and 138 of the *S*. *dysgalactiae* subsp. *equisimilis* isolates (from 135 patients) were sequenced to identify the *emm* gene.

Routine blood samples were drawn into aerobic and anaerobic bottles and cultivated by standard methods as reported (*8*). *S.* *dysgalactiae* subsp. *equisimilis* isolates were further analyzed by *emm* typing. Nontypeable strains and strains isolated from patients with recurrent bacteremia were characterized by using pulsed-field gel electrophoresis (PFGE).

The *emm* typing was performed according to the protocol of the Centers for Disease Control and Prevention (www.cdc.gov/ncidod/biotech/strep/strepblast.htm). If the *emm* gene could not be amplified with primers 1 and 2, alternative primers MF1/MR1 were used (*9*). PFGE was performed as described (*10*). DNA profiles were analyzed by using Bionumerics software (Applied Maths, Kortrijk, Belgium) and interpreted according to the guidelines described (*11*). Strains with >85% similarity were considered to be related types.

SPSS software version 7.5 (SPSS, Chicago, IL, USA) was used for statistical analyses, and a 2-sided p value <0.05 was regarded as the level for significance. Categorical data were analyzed by χ² test or Fisher exact test as appropriate. Nonparametric data were analyzed by using the Mann-Whitney U test. Odds ratios were expressed with 95% confidence intervals.

**Figure 1:**
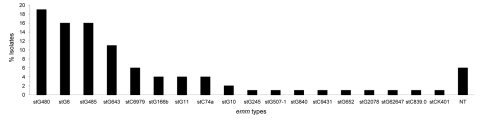
*emm* types of 138 *Streptococcus dysgalactiae* subsp. *equisimilis* bacteremic isolates obtained during 1995–2004, Finland. NT, nontypeable.

The median age of patients (73 men, 62 women) was 67 years (range 17–90 years). Cardiovascular diseases (41%), diabetes (25%), and malignancies (23%) were the 3 most prominent underlying conditions. We found 18 *emm* types (including 4 subtypes of stG6: stG6.0, stG6.1, stG6.3, and stG6.4). StG480 (27 isolates), stG6 (23 isolates), and stG485 (22 isolates) were the 3 most common *emm* types and represented 51% of all isolates (Figure 1). Eight of group G *S*. *dysgalactiae* subsp. *equisimilis* isolates remained nontypeable. PFGE analysis showed 2 strains to be related (>85% similarity). The rest of the nontypeable strains were sporadic (6 isolates).

**Figure 2:**
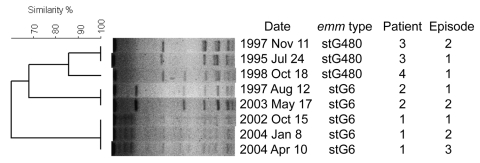
Dendrogram and pulsed-field gel electrophoresis (PFGE) profiles of the strains isolated from patients with recurrent group G *Streptococcus* *dysgalagtiae* subsp. *equisimilis* bacteremia, Finland. Dendogram was generated by using Bionumerics software (Applied Maths, Kortrijk, Belgium) with a 1.0% lane optimization and 1.5% band position tolerance.

We divided bacteremia episodes into 2 groups: those caused by the 5 most common *emm* types and each representing >5% of all episodes (97 episodes, common *emm* types) and those caused by the less common or nontypable *emm* types (41 episodes, rare *emm* types). We could not find an association between *emm* type and clinical features such as age or underlying disease. Severe disease was caused more often by rare *emm* types than by common *emm* types. Mortality rates were higher in patients with bacteremia caused by rare types than that caused by common types (Table 1). Four patients had recurrent *S*. *dysgalactiae* subsp. *equisimilis* bacteremia (Table 2). PFGE profiles showed that strains isolated from the same patient in recurring infections were identical (Figure 2).

**Table 1:** Disease severity among 138 episodes of *Streptococcal* *dysgalactiae* subsp. *equisimilis* bacteremia, Finland, 1995–2004*

Disease severity	No. (%) common *emm* types, n = 97	No. (%) rare *emm* types, n = 41	Odds ratio (95% CI)	p value†
30-day mortality rate	11 (11)	12 (29)	3.2 (1.3–8.1)	0.01
Patient admitted to ICU	5 (5)	6 (15)	3.2 (0.9–11)	0.084
Patient death or ICU treatment	12 (12)	15 (37)	4.1 (1.7–9.8)	0.001
Hypotension‡	13 (13)	10 (24)	2.1 (0.8–5.2)	0.113
DIC§	2 (2)	6 (15)	8.1 (1.6–42.3)	0.009
Multiorgan failure	2 (2)	4 (10)	5.1 ( 0.9–29.2)	0.064
STSS¶	2 (2)	4 (10)	5.1 ( 0.9–29.2)	0.064

*CI, confidence interval; ICU, intensive care unit; DIC, disseminated intravascular coagulation; STSS, streptococcal toxic shock syndrome. Patients who had both clinical data and isolates available. †χ² test or Fisher exact test as appropriate. ‡Hypotensive (BP <90 mm Hg) at least once 0–2 days after positive blood culture. §Thrombocyte count <100 x 10^9^/L. ¶The definition of STSS was based on a consensus definition, including identification of β-hemolytic streptococci from a normally sterile site, septic shock, and multiorgan failure.

**Table 2:** Characteristics of recurrent episodes of group G *Streptococcal* *dysgalactiae* subsp. *equisimilis* bacteremia, Finland, 1995–2004*

Patient no.	*emm* type			Time to recurrence, mo	Clinical signs	PFGE pattern
	Episode 1	Episode 2	Episode 3			
1	stG6	stG6	stG6	15; 3	Cellulitis	Unique, identical in episodes 1–3
2	stG6	stG6		68	Cellulitis	Unique, identical in episodes 1 and 2
3	stG480†	stG480		28	Spondylitis	Unique, identical in episodes 1 and 2
4	stG480	NA‡		21	Cellulitis	Unique

*PFGE, pulsed-field gel electrophoresis. †Blood culture taken outside Pirkanmaa Health District, isolate available. ‡Blood culture taken outside Pirkanmaa Health District, no isolate available.

Common *emm* types were more frequently manifested as skin and soft tissue infections than were rare *emm* types, 75% vs. 54%, respectively (p = 0.012). The most frequent source of bacteremia was cellulitis (51%). We also found an association between a common *emm* type and cellulitis. Cellulitis was a more frequent clinical sign among patients with infections caused by common *emm* types than by rare *emm* types (p = 0.007); 64% of patients infected by common *emm* types had cellulitis as an initial clinical manifestation versus 39% of patients infected by rare *emm* types.

## Conclusions

Our study showed that mortality rates were higher in patients with *S. dysgalactiae* subsp. *equisimilis* bacteremia caused by rare *emm* types than in those with bacteremia caused by common *emm* types. The reason for this finding is unclear. One explanation for this might be that patients contract certain prevailing bacterial strains *(*so-called common types) more often, and a prior antigen challenge and subsequent humoral response may play a role. Severe disease (death or intensive care unit treatment) was also caused more often by rare *emm* types than by common *emm* types. We found also an association between a common *emm* type and cellulitis as a clinical manifestation; the common *emm* types were also associated with skin and soft tissue infections.

In our comprehensive study with molecular typing data for 138 invasive *S*. *dysgalactiae* subsp. *equisimilis* isolates from human infections, we found 18 *emm* types, which is consistent with previous reports by Cohen-Poradosu et al. (*12*) and Broyles et al. (*13*) *.* These 2 studies reported stG485.0. or StG6, StG245, and StG2078 as the most common *emm* types, respectively. Thus, *emm* typing provides a useful tool for comparative epidemiologic analysis of GGS isolates from various geographic regions. Our results also suggest that certain *emm* types may prevail among bacteria that cause human infections. Our study did not show any obvious time shifts in the occurrence of certain *emm* types.

A noteworthy finding in our series was the high frequency of recurrent group G *S*. *dysgalactiae* subsp. *equisimilis* bacteremia as reported earlier (*12*,*14*). Clinicians should be alert to this phenomenon, which seems to be more common than recurrent group A streptococcal bacteremia.

The dynamics of interspecies transfer of virulence loci between group A streptococci, GGS, and GCS (*3*), as well as potential genetic transfer or intragenomic events causing interconversion of group antigen types, remains to be resolved. Further characterization of the strains by multilocus sequence typing would be of interest (*15*).

We conclude that severity of disease and mortality rates were higher in persons with *S*. *dysgalactiae* subsp. *equisimilis* bacteremia caused by rare *emm* types than that caused by common *emm* types. Skin and soft tissue infections such as cellulitis were significantly more frequent clinical signs among episodes caused by common *emm* types.
